# Association between intimate partner violence and leukocyte telomere length: a retrospective cohort study of 144 049 UK Biobank participants

**DOI:** 10.1017/S2045796023000112

**Published:** 2023-04-24

**Authors:** Ko Ling Chan, Camilla K. M. Lo, Xiao-Yan Chen, Patrick Ip, Wing Cheong Leung, Paul G. Shiels, Jill P. Pell, Helen Minnis, Frederick K. Ho

**Affiliations:** 1 Department of Applied Social Sciences, The Hong Kong Polytechnic University, Hung Hom, Hong Kong; 2 Department of Paediatrics and Adolescent Medicine, The University of Hong Kong, Pokfulam, Hong Kong; 3 Department of Paediatrics and Adolescent Medicine, Hong Kong Children's Hospital, Hong Kong, Hong Kong; 4 Department of Obstetrics & Gynaecology, Kwong Wah Hospital, Hong Kong, Hong Kong; 5 School of Cancer Sciences, University of Glasgow, Glasgow, UK; 6 School of Health and Wellbeing, University of Glasgow, Glasgow, UK

**Keywords:** Depression, intimate partner violence, PTSD, telomere length

## Abstract

**Aims:**

Intimate partner violence (IPV) is a public health challenge negatively affecting victims’ health. Telomere length (TL), a marker for biological ageing, might be reflective of the mechanisms through which IPV leads to adverse health outcomes. The objective of the current study was to explore the association between IPV and leucocyte TL.

**Methods:**

We conducted an analysis using a subset of the UK Biobank (*N* = 144 049). Physical, sexual and emotional IPV were reported by the participants. DNA was extracted from peripheral blood leukocytes. TL was assayed by quantitative polymerase chain reaction. We used multivariable linear regressions to test the associations between IPV and TL adjusted for age, sex, ethnicity, deprivation, education, as well as symptoms of depression and post-traumatic stress disorder in a sensitivity analysis.

**Results:**

After adjusting for sociodemographic factors, any IPV was associated with 0.02-s.d. shorter TL (*β* = −0.02, 95% CI −0.04 to −0.01). Of the three types of IPV, physical violence had a marginally stronger association (*β* = −0.05, 95% CI −0.07 to −0.02) than the other two types. The associations of numbers of IPV and TL showed a dose–response pattern whereby those who experienced all three types of IPV types had the shortest TL (*β* = −0.07, 95% CI −0.12 to −0.03), followed by those who experienced two types (*β* = −0.04, 95% CI −0.07 to −0.01). Following additional adjustment for symptoms of depression and PTSD, the associations were slightly attenuated but the general trend by number of IPVs remained.

**Conclusions:**

Victims of IPV, particularly those exposed to multiple types of IPVs, had shorter TL indicative of accelerated biological ageing. Given that all three types of IPV are linked to TL, clinical practitioners need to comprehensively identify all types of IPV and those who received multiple types. Further studies should explore the association of violence with changes in TL over time, as well as to which extent biological ageing is a mechanistic factor.

## Introduction

Intimate partner violence (IPV), which encompasses physical, sexual and emotional violence perpetrated by an intimate partner or ex-partner, has been recognised as a pervasive public health problem (World Health Organization, 2018). Globally, nearly 30% of women aged 15 years and older have been exposed to IPV (World Health Organization, 2018). Mounting research has shown the detrimental consequences of IPV on victims’ health and well-being, in particular depression and post-traumatic stress disorder (PTSD) (Lagdon *et al*., 2014; Bacchus *et al*., 2019).

The growing field of biological markers such as telomere length (TL) has opened a unique avenue for understanding the biological mechanisms underpinning diseases (Ridout *et al*., 2018). Telomeres are nucleon–protein complexes at the end of the eukaryotic chromosomes. The DNA component, comprising (TTAGGG)n, is a biomarker of ageing and adverse health outcomes that shortens in somatic cells as they replicate (Blackburn *et al*., 2015; Wang *et al*., 2018). Multiple Mendelian randomisation studies have shown the potential causal effect of telomere shortening and a number of health outcomes, such as cancer and cardiovascular disease (Haycock *et al*., 2017; Kuo *et al*., 2019). The existing literature on the relationship between traumatic events and TL has mainly focused on early life (Hanssen *et al*., 2017; Ridout *et al*., 2018). Studies among adults on the relationship between IPV and TL have produced inconsistent findings. For example, Humphreys *et al*. (2012) found that TL was significantly shorter in 61 formerly abused women than in 41 controls. However, such an association was not found in another 30-year birth cohort study of 677 women (Jodczyk *et al*., 2014). Interindividual variation in TL at any given chronological age remains substantial and is often a complicating factor in such studies, as are exposome features (i.e. biotic and abiotic life course exposures) (Shiels *et al*., 2021). Additionally, these studies did not specify the types of IPV and their separate and combined effects, nor consider mental health confounders. Thus, the mixed findings may be due to insufficient power or confounding. Research shows experiencing multiple forms of victimisation (Chan *et al*., 2021), such as more than one type of IPV (Lagdon *et al*., 2014), can increase the severity of mental health outcomes, studies on TL require greater granularity in their measurement of the types and dosage of IPV.

Hence, this study aimed to examine the association between IPV types (physical, sexual, emotional) and TL in a sufficiently large sample size from the UK Biobank (UKB). This allows mental health symptoms and other covariates to be taken into consideration in the analyses. The large sample also enabled the relative importance of the multiple exposures to various types of IPV on TL to be compared.

## Methods

### Study design and participants

UK Biobank recruited more than 502 506 participants (aged 40–69 years) from the general population between 2006 and 2010. Participants were assessed at one of 22 assessment centres across England, Scotland and Wales. They completed a self-administered questionnaire and a face-to-face interview. The details of the study design and protocols of UK Biobank are provided elsewhere (Sudlow *et al*., 2015). This is a retrospective cohort study consisting of the subsample of UK Biobank participants who completed a web-based mental health questionnaire, in which they reported any exposure to IPV since 16 years of age as well as symptoms of depression and PTSD. TL measurements were undertaken on the DNA of participants’ blood samples. UK Biobank received ethical approval from the North-West Multi-centre Research Ethics Committee (reference 11/NW/0382) and all participants provided written informed content.

### Measures

#### Exposure: IPV

IPV was self-reported through an online questionnaire using a five-point Likert scale for each of three types of IPV (physical, emotional and sexual violence) that occurred since the age of 16 years. The items were adapted from the British Crime Survey (Khalifeh *et al*., 2015). The threshold values on the Likert scale were used to define the presence (‘Sometimes’, ‘Often’, ‘Very often’) or absence (‘Never’ and ‘Rarely’) of each type of IPV. The categorisation is based on the assumption that people who reported ‘rarely’ did not have chronic, repeated exposure to violence. The exposure variable was the number of types of IPV, and was categorised as 0, 1, 2 and 3. Detailed descriptions of the variables are contained in Supplemental Table 1.

#### Outcome: leukocyte telomere length

Detailed information on the measurement of TL in UKB has been provided elsewhere (Codd *et al*., 2022). Briefly, DNA was extracted from peripheral blood leukocytes. TL was assayed using the quantitative polymerase chain reaction. The assay results were presented as a relative ratio of the telomere repeat copy number (T) to a single-copy gene (S). The calculated T/S ratios were then adjusted for technical variation, log-transformed and *Z*-standardised to approximate a normal distribution with a mean of 0 and s.d. of 1. Over 23 000 measurements had undergone a reproducibility check which resulted in good coefficient of variation (median 5.53, interquartile range 2.67–9.68) (Codd *et al*., 2022). Technicians who underwent the TL assessment had no access to the participants’ other data, including exposure to violence.

#### Other variables

The online questionnaire also measured current symptoms of depression and PTSD, using two well-established tools: the Patient Health Questionnaire-9 (PHQ-9), and the Post-traumatic stress disorder Check List – civilian Short version (PCL-S). Specifically, PHQ-9 measures depression severity from the frequency of nine items, ranging from 0 (not at all) to 3 (nearly every day). All items were summated to provide a total score of depression severity, with higher scores indicating more symptoms. Previous work has demonstrated the validity and reliability of using this scale in UK Biobank (Kandola *et al*., 2020). PCL-S consists of five items that map onto the DSM-IV criteria (Wilkins *et al*., 2011).

Demographic information was collected, including sex, age, ethnicity, highest educational level and Townsend Deprivation Index; a composite area-based measure derived from unemployment, car ownership, household overcrowding and owner occupation, with higher scores indicating higher levels of deprivation (Elovainio *et al*., 2017; Howe *et al*., 2020).

### Data analyses

Descriptive statistics were first computed to describe the participants’ characteristics, IPV experiences and mental health problems. A set of independent *χ*^2^ tests were performed to compare the potential sex differences in categorised variables (ethnicity, education attainment, IPV types and numbers of IPV (i.e. 0, 1, 2 and 3)) and *t-*tests were performed to compare continuous variables (age, deprivation index, mental health symptoms and TL). We then used *χ*^2^ and *t-*tests to compare the possible differences in demographic characteristics, mental health problems and TL by the number of IPV.

To explore the association between IPV and TL, we conducted multivariable linear regression models, adjusted for sociodemographic characteristics: age, sex, ethnicity, deprivation index and education. These covariates were adjusted because they are likely to be confounders, affecting the likelihood of IPV as well as TL. Both the types of IPV and the number of types were used as exposure variables. Symptoms of depression and PTSD were additionally adjusted in a separate sensitivity analysis. These are possible confounders (as depression and PTSD might affect the recall of IPV) or mediators (as IPV could increase the risk of depression and PTSD) which we do not have data in this study to ascertain. The sensitivity analysis would be subject to overadjustment bias if depression and PTSD are, in fact, mediators. Other chronic conditions were not adjusted in the analysis because they are less likely to affect the likelihood or the report of IPV. Finally, we investigated the potential interaction between demographic characteristics and IPV on TL. All statistical analyses were performed using R version 4.0.2. *p* < 0.05 in two-sided tests was considered statistically significant.

## Results

Of 502 488 UK Biobank participants, 156 379 (31.1%) completed all the IPV-related questions. Of these, 8780 (5.6%) and 3550 (2.4%) were excluded due to no valid TL and covariate data, respectively. Therefore, the sample size was 144 049 (Supplementary Fig. 1).

Table 1 shows the participants’ characteristics by sex. The mean age of the participants was 55.89 (s.d. 7.74) years, 56.25% were female and 16.16% reported experience of IPV. The most frequently reported type was emotional (14.16%), followed by physical (6.37%) and then sexual violence (2.52%). Overall, 10.68, 4.07 and 1.41% of participants reported one, two and three types, respectively. Compared with men, women reported significantly more IPV events across all three types of IPV.

**Table 1.** Participants’ characteristics by sex

	Overall	Female	Male	*p*-value
Total *n*	144 049	81 027	63 022	
Age, mean (SD)	55.89 (7.74)	55.40 (7.66)	56.52 (7.80)	<0.0001
Ethnicity				<0.0001
White	140 030 (97.21)	78 743 (97.18)	61 287 (97.25)	
South Asian	1171 (0.81)	519 (0.64)	652 (1.03)	
Black	972 (0.67)	569 (0.70)	403 (0.64)	
Chinese	330 (0.23)	228 (0.28)	102 (0.16)	
Mixed	759 (0.53)	488 (0.60)	271 (0.43)	
Any other	787 (0.55)	480 (0.59)	307 (0.49)	
Deprivation index, mean (SD)	−1.72 (2.83)	−1.68 (2.82)	−1.77 (2.84)	<0.0001
College or University degree	65 847 (45.71)	35 872 (44.27)	29 975 (47.56)	<0.0001
PHQ-9, mean (SD)	2.75 (3.68)	3.01 (3.79)	2.42 (3.51)	<0.0001
PCL-S, mean (SD)	1.70 (2.77)	1.96 (2.92)	1.37 (2.52)	<0.0001
LTL, Log(T/S ratio), *z*-score, mean (SD)	0.05 (0.99)	0.14 (0.99)	−0.06 (0.99)	<0.0001
Any IPV	23 274 (16.16)	17 906 (22.10)	5368 (8.52)	<0.0001
Physical	9174 (6.37)	7439 (9.18)	1735 (2.75)	<0.0001
Emotional	20 395 (14.16)	15 802 (19.50)	4593 (7.29)	<0.0001
Sexual	3623 (2.52)	3474 (4.29)	149 (0.24)	<0.0001
Number of IPV types				<0.0001
0	120 775 (83.84)	63 121 (77.90)	57 654 (91.48)	
1	15 384 (10.68)	11 064 (13.65)	4320 (6.85)	
2	5862 (4.07)	4875 (6.02)	987 (1.57)	
3	2028 (1.41)	1967 (2.43)	61 (0.10)	

*Note*. LTL, leucocyte telomere length. LTL was expressed in T/S ratio here and in further analyses.

Table 2 shows differences in demographic characteristics, mental health problems and TL in multiple types of IPV (i.e. 0, 1, 2 and 3). Those who experienced more IPV types were younger, more likely to be female, South Asian, Black, or with mixed ethnicity, had lower highest education level and higher levels of deprivation. Exposure to higher numbers of IPV was also associated with higher levels of depression and PTSD.

**Table 2.** Participants’ characteristics by sex by number of IPV types

	Number of IPV types				
	0	1	2	3	*p*-value
Total *n*	121 089	15 475	5860	2012	
Age, mean (SD)	56.17 (7.73)	54.73 (7.63)	53.87 (7.67)	53.99 (7.36)	<0.0001
Sex					<0.0001
Female	63 121 (52.3)	11 064 (71.9)	4875 (83.2)	1967 (97.0)	
Male	57 654 (47.7)	4320 (28.1)	987 (16.8)	61 (3.0)	
Ethnicity					<0.0001
White	117 500 (97.3)	14 962 (97.3)	5650 (96.4)	1918 (94.6)	
South Asian	990 (0.8)	101 (0.7)	53 (0.9)	27 (1.3)	
Black	780 (0.6)	94 (0.6)	61 (1.0)	37 (1.8)	
Chinese	285 (0.2)	30 (0.2)	12 (0.2)	3 (0.1)	
Mixed	563 (0.5)	116 (0.8)	55 (0.9)	25 (1.2)	
Any other	657 (0.5)	81 (0.5)	31 (0.5)	18 (0.9)	
Deprivation index, mean (SD)	−1.82 (2.77)	−1.36 (2.98)	−0.96 (3.15)	−0.47 (3.23)	<0.0001
College or University degree	55 805 (46.2)	7048 (45.8)	2272 (38.8)	722 (35.6)	<0.0001
PHQ-9, mean (SD)	2.41 (3.29)	4.28 (4.60)	4.81 (5.09)	5.85 (5.96)	<0.0001
PCL-S, mean (SD)	1.39 (2.39)	3.02 (3.56)	3.66 (3.98)	4.78 (4.60)	<0.0001
LTL, z-score, mean (SD)					
38–50 years female	0.33 (0.98)	0.30 (0.99)	0.26 (0.96)	0.23 (0.94)	0.001
51–60 years female	0.16 (0.98)	0.14 (0.99)	0.15 (0.94)	0.06 (0.93)	0.02
61–72 years female	−0.05 (0.98)	−0.05 (0.96)	−0.09 (0.95)	−0.04 (1.00)	0.49
38–50 years male	0.22 (0.98)	0.23 (0.97)	0.19 (0.98)	0.10 (0.97)	0.80
51–60 years male	−0.05 (0.96)	−0.02 (0.95)	−0.07 (0.93)	−0.50 (0.70)	0.07
61–72 years male	−0.26 (0.97)	−0.28 (1.00)	−0.28 (1.03)	−0.28 (1.59)	0.93

**Fig. 1.**
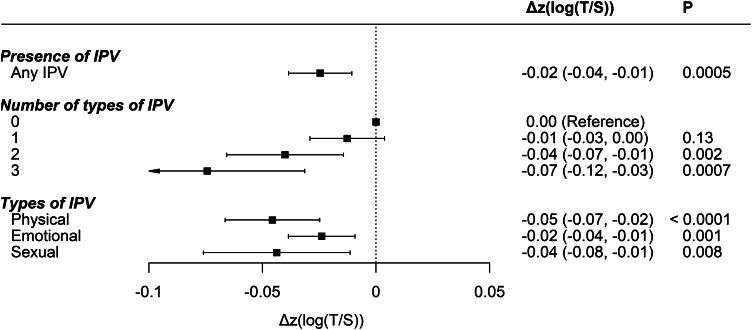
Association between IPV and telomere length. *Note*. Adjusted for age, sex, ethnicity, deprivation and education.

After adjusting for sociodemographic factors, any IPV was associated with 0.02-s.d. shorter TL (*β* = −0.02, 95% CI −0.04 to −0.01) (Fig. 1). Of the three types of IPV, physical violence had a marginally stronger association (*β* = −0.05, 95% CI −0.07 to −0.02) than the other two types. The associations of numbers of IPV and TL showed a dose–response pattern whereby those who experienced all three types of IPV types had the shortest TL (*β* = −0.07, 95% CI −0.12 to −0.03), followed by those who experienced two types (*β* = −0.04, 95% CI −0.07 to −0.01). Following additional adjustment for symptoms of depression and PTSD, the associations were slightly attenuated but the general trend by number of IPVs remained (Table 3).

**Table 3.** Association between IPV and standardised LTL

	*β* (95% CI)	*p*
Any IPV	−0.02 (−0.03, 0.00)	0.02
Number of IPV		
1	−0.01 (−0.02, 0.01)	0.50
2	−0.03 (−0.06, −0.01)	0.02
3	−0.06 (−0.11, −0.02)	0.005
Types of IPV		
Physical	−0.04 (−0.06, −0.02)	0.0004
Emotional	−0.02 (−0.03, 0.00)	0.048
Sexual	−0.03 (−0.07, 0.00)	0.04

*Note*. Adjusted for age, sex, ethnicity, deprivation, education and symptoms for depression and PTSD.

Table 4 shows the associations between IPV and TL by socio-demographic subgroups. The association was stronger among younger participants, particularly in the 38–60 years age group (*P*_interaction_ = 0.01). No interactions reached statistical significance for other demographic characteristics.

**Table 4.** Association between IPV and standardised LTL by sociodemographic subgroups

	*β* (95% CI)	*p*	*P*_interaction_
Sex			0.12
Female	−0.02 (−0.03, −0.01)	<0.0001	
Male	−0.01 (−0.03, 0.01)	0.29	
Age			0.01
38–50	−0.02 (−0.04, −0.01)	0.002	
51–60	−0.02 (−0.03, 0.00)	0.03	
61–72	−0.01 (−0.03, 0.01)	0.18	
Ethnicity			0.89
White	−0.02 (−0.03, −0.01)	<0.0001	
Non-white	−0.04 (−0.09, 0.01)	0.09	
Deprivation index			0.61
Less deprived	−0.02 (−0.03, 0.00)	0.01	
More deprived	−0.02 (−0.03, −0.01)	<0.0001	
Education attainment			0.89
With university degree	−0.02 (−0.04, −0.01)	0.002	
Without university degree	−0.02 (−0.03, −0.01)	0.0007	

*Note*. Adjusted for age, sex, ethnicity, deprivation, education and symptoms for depression and PTSD.

## Discussion

### Main findings

This is among the first studies which clearly show that each type of IPV was associated with shorter TL and the association was the strongest among participants who experienced multiple types of IPV, albeit with a small effect size. The findings were consistent even when adjusted for symptoms of depression and PTSD, which could affect the recall of IPV or may be a mediator of the association between IPV and TL. Given that shorter TL is associated with various major illnesses, within the ‘diseasome of ageing’ (Shiels *et al*., 2017, 2021), including cardiovascular disease, chronic kidney disease, cancer and Alzheimer's disease (Haycock *et al*., 2017), TL could reflect a biological mechanism on the burden of physiological ‘wear and tear’. This suggests that the IPV could accelerate biological ageing, which predisposes to future adverse health outcomes.

### Findings in the context of existing evidence

In this study, we found that women were more likely to report all three types of IPV. Previous research reported mixed findings on gender and IPV victimisation (Chan, 2011), which may be related to gender-specific factors and cultural-specific gender roles (Chan, 2011; Laskey *et al*., 2019). The traditional gendered (or feminist) perspective suggests that the perpetrator of IPV is male and the victim is female (Martín-Fernández *et al*., 2018). However, men's physio-psychological response to IPV may be similar to that of women. The gender differences in IPV prevalence observed in our study may result from different coping strategies (Laskey *et al*., 2019), such that male victims are less likely to report even if they experience similar victimisation. Regardless, given the evidence on the association between IPV and TL both among the victims (as shown in this study) and among the offspring (Chan *et al*., 2019), future studies should look into whether TL is a mechanistic factor leading to health outcomes, or whether TL can be a marker to help risk stratify IPV victims for health-related interventions.

We found the association between IPV and TL was stronger in younger participants. Two competing theories have addressed how the relationship might change with age. Cumulative disadvantage theory states that adverse events over the lifespan could produce greater health risks in later life (Dannefer, 2003; Ferraro and Shippee, 2009). So, the inverse relationship between IPV and TL should become stronger across the lifespan. Indeed, a cohort of women aged 35–74 years who had a sister with breast cancer found an association between perceived stress and shorter TL only among women aged 55 years and older (Parks *et al*., 2009). Conversely, the age-as-leveller theory argues that inequalities in health reduce across the lifespan (House *et al*., 1994; Lauderdale, 2001). Studies support the age-as-leveller position that stressful life events were inversely associated with TL in adults aged 22–44 but not in adults aged 45–69 (McFarland *et al*., 2018). Our current results are consistent with the age-as-leveller theory. Older adults may be more likely to report age-related diseases such as cardiovascular diseases (Gruber *et al*., 2021) that are associated with TL shortening. These diseases may cover up the independent influence of psychosocial stressors (Schaakxs *et al*., 2016). This, however, seems counterintuitive as TL shortening is reflective of an accumulation of ‘wear and tear’ from a range of exposome features that act both cumulatively, synergistically and independently (Dai *et al*., 2021; Mafra *et al*., 2021). Another possibility is that older adults with the most damaged TL may not participate in the study because of health issues or death (Schaakxs *et al*., 2016); this healthy survivor effect without subjects with the most shortened TL might underestimate the true relationship between IPV and TL. Given these explanations are tentative and the nature of retrospective design, future studies with more robust methodologies including serial measurement of TL are needed to further explore this topic.

The present study underscores some complexities of research into IPV and TL. First, each type of IPV was negatively associated with TL and the association was strongest in physical violence. Existing studies on the relationship between IPV and TL found inconsistent results (Humphreys *et al*., 2012; Jodczyk *et al*., 2014), and very few further investigated the associations of different and multiple types of IPV with TL. The influence of different types of violence in early life on TL may provide insights into understanding the issue and show inconsistent results. Vincent *et al*. (2017) found no associations between physical/sexual/emotional violence and TL from a UK sample (ages 20–84). However, a study of 1135 women (Mason *et al*., 2015) has found stronger evidence of an association between physical violence and TL than sexual violence, consistent with our findings. Our current study extends previous work exploring the influence of all three types of IPV. All three types of IPV showed negative relationships with TL in unadjusted models, suggesting the importance of comprehensively evaluating all three types of IPV.

Secondly, there was a dose–response relationship between number of types of IPV and TL. This finding echoes the allostatic load model that highlights the cumulative impact of multiple sources of stress (Danese and McEwen, 2012). This notion was also evidenced in the influence of stressful childhood events on TL, with a relatively consistent result showing a critical role of cumulative childhood stress on TL (Shalev *et al*., 2013; Puterman *et al*., 2016). Researchers of adulthood stress also call for investigations on cumulative stressors (van Ockenburg *et al*., 2015; Verhoeven *et al*., 2015). Our current findings suggest that identifying victims with multiple types of IPV might be beneficial if system-level interventions are in place (Hamberger *et al*., 2015). Additional research is needed to explore further this critical issue, including assessing IPV duration and severity.

### Strengths and limitations

One of the strengths of this study is the use of a relatively large sample using UKB (*N* = 144 049) of both sexes, providing sufficient power to detect differences. In addition, we were able to explore the separate and combined effects of different types of IPV and, therefore, demonstrate a dose–response relationship. Certain limitations and considerations should be acknowledged. Firstly, the exposure to IPV was recalled retrospectively and therefore may have been subject to recall bias. The IPV measurement also has no metrics of validity and reliability. As a result of the potential reporting bias and residual confounding, we should not interpret causality from the findings of this study. Secondly, there were minimal data on the duration, frequency and severity of violence in the data. Thirdly, TL was only available as T/S ratio rather than differences in base pairs. Fourthly, with the small effect size, some tests, particularly in subgroup analysis, could have been underpowered. Last but not least, UK Biobank is not representative of the general UK population with a healthy volunteer bias, particularly among those who completed the online mental health survey (Ho *et al*., 2020). This could explain the stronger association in younger age group and distort the association estimates if the participation was caused by both IPV and TL, even though it does not appear likely. Previous analysis has shown that the association estimates from the UK Biobank are comparable to that from population-representative cohorts (Batty *et al*., 2020).

### Implications

The significant relationship between IPV and TL has real-world implications. Given that all three types of IPV are linked to TL, clinical practitioners need to comprehensively identify all types of IPV, especially physical IPV which had the strongest individual association, and multiple IPV which had the strongest overall association. Importantly, these provide further evidence for prioritising intervention for those individuals. While causality cannot be established from this study, future studies should examine whether TL is a mechanism and/or a risk marker, which would provide the basis to use TL as a prognostic factor in clinical practice.

## Conclusions

IPV was associated with TL in a dose–response pattern. Further studies should explore the association of violence with changes in TL over time, as well as to which extent these translate to adverse health outcomes.

## Data Availability

The data can be requested from the UK Biobank (https://www.ukbiobank.ac.uk/).

## Supplementary material

For supplementary material accompanying this paper visit http://doi.org/10.1017/S2045796023000112. click here to view supplementary material
