# Supplementary material for: Association between intimate partner violence and leukocyte telomere length: a retrospective cohort study of 144 049 UK Biobank participants
Source: Epidemiol Psychiatr Sci. 2023 Apr 24;32:e26. doi: 10.1017/S2045796023000112 (PMC10130734; doi:10.1017/S2045796023000112)
Supplement: Supplementary file 1 [file S2045796023000112sup001.docx]

# Supplemental Appendix

## Supplementary Table 1. Descriptions of questionnaire items

| **Scale** | **Variable item** | **Item** | |
| --- | --- | --- | --- |
| **Exposure -** Intimate partner violence (IPV) | | | |
| IPV - Partner physical violence | Since I was sixteen... A partner or ex-partner deliberately hit me or used violence in any other way | *Original item:*  Never  Rarely  Sometimes  Often  Very often | *Recoded as:*  Absence  Absence  Presence  Presence  Presence |
| IPV - Partner emotional violence | Since I was sixteen... A partner or ex-partner repeatedly belittled me to the extent that I felt worthless |  |  |
| IPV - Partner sexual violence | Since I was sixteen... A partner or ex-partner sexually interfered with me, or forced me to have sex against my wishes |  |  |
| **Covariates -** Mental health symptoms | | | |
| **Depressive symptoms** (9 items)  Patient Health Questionnaire 9-question version (PHQ-9) | Over the last 2 weeks, how often have you been bothered by any of the following problems:   1. Little interest or pleasure in doing things 2. Feeling down, depressed, or hopeless 3. Trouble falling or staying asleep, or sleeping too much 4. Feeling tired or having little energy 5. Poor appetite or overeating 6. Feeling bad about yourself or that you are a failure or have let yourself or your family down 7. Trouble concentrating on things, such as reading the newspaper or watching television 8. Moving or speaking so slowly that other people could have noticed? Or the opposite – being so fidgety or restless that you have been moving around a lot more than usual 9. Thoughts that you would be between off dead or of hurting yourself in some way | 0 = Not at all  1 = Several days  2 = More than half the days  3 = Nearly every day | |
| **PTSD symptoms (**5 items)  Post-traumatic stress disorder Check List – civilian Short version (PCL-S) | Please indicate how much you have been bothered by that problem in the past month:   1. Repeated, disturbing memories, thoughts, or images of a stressful experience? 2. Feeling very upset when something reminded you of a stressful experience? 3. Avoiding activities or situation because they reminded you of a stressful experience?   Please indicate how much you have been bothered by that problem in the past month:   1. Feeling distant or cut off from other people? 2. Feeling irritable or having angry outbursts? | 0 = Not at all  1 = A little bit  2 = Moderately  3 = Quite a bit  4 = Extremely | |

## Supplementary Figure 1. Participant flowchart

502,488 UK Biobank participants

N = 156,379

N = 147,599

144,049 included in the analysis

346,109 excluded due to no valid IPV data

8,780 excluded due to no valid telomere length data

3,550 excluded due to no valid covariate data
